# Sevoflurane-induced POCD-associated exosomes delivered miR-584-5p regulates the growth of human microglia HMC3 cells through targeting BDNF

**DOI:** 10.18632/aging.204398

**Published:** 2022-11-30

**Authors:** Jia Zhao, Wei Zhang, Shentong Wang, Zinan Li, Yuqi Huang, Longyun Li

**Affiliations:** 1 Department of Anesthesiology, China-Japan Union Hospital of Jilin University, Changchun 130033, Jilin, China

**Keywords:** post operative cognitive dysfunction, sevoflurane, HMC3 cells, exosomes, miR-584-5p/BDNF

## Abstract

Background: Inhalation of sevoflurane can cause neuronal apoptosis, and cognitive disorders, inducing to the occurrence and progression of post operative cognitive dysfunction (POCD). This study aimed to explore the roles of sevoflurane-induced POCD-associated exosomes on HMC3 cells and its related mechanisms.

Methods: Exosomes were isolated from the plasma of sevoflurane-induced POCD or non-POCD patients, and were then sent for small RNA sequencing. Real-time quantitative PCR (RT-qPCR) was used to verify the sequencing results, and miR-584-5p was chosen for subsequent study. HMC3 cells were respectively transfected with POCD-derived exosomes and miR-584-5p mimics, and cell viability and apoptosis were measured. Dual-luciferase reporter gene assay was applied to confirm the target of miR-584-5p.

Results: After sequencing, 301 differentially expressed miRNAs were identified, including 184 up-regulated miRNAs and 117 down-regulated miRNAs, and were significantly enriched in 3577 GO terms and 121 KEGG pathways. Due to the high level of miR-584-5p in sevoflurane-treated POCD-derived exosomes, HMC3 cells with miR-584-5p enrichment were successfully established. Compared with the control group, POCD-derived exosomes and miR-584-5p significantly inhibited viability and promoted apoptosis of HMC3 cells (*P* < 0.05). The IL-1β and TNF-α levels were significantly increased after POCD-derived exosomes and miR-584-5p mimics treatment compared to the control group (*P* < 0.05). Besides, POCD-derived exosomes and miR-584-5p mimics significantly down-regulated the expression levels of BDNF and p-TrkB, and up-regulated Caspase 3 and IL-1β. Finally, BDNF was confirmed to be the target of miR-584-5p.

Conclusions: Sevoflurane-induced POCD-associated exosomes delivered miR-584-5p may regulate the growth of HMC3 cells via targeting BDNF.

## INTRODUCTION

Postoperative delirium, a mysterious clinical syndrome, is also called post operative cognitive dysfunction (POCD) that manifested by disturbance of consciousness, disordered behavior, aimlessness, and inability to concentrate [1]. Usually, the onset of delirium is acute accompanied with a fluctuating course. Delirium often occurs in the elderly, especially when there are pre-existing neurocognitive impairment and following insults such as infections or trauma [2]. Since many vulnerable older adults require surgery, POCD particularly is a growing public health problem, occurring in 20%-50% of people older than 60 years old after major surgery [3, 4]. In addition, POCD is also related to the increased mortality, decreased cognition and function, longer hospital stays, and significant annual medical costs [5, 6]. More and more evidence show that the pathogenesis of POCD involved a variety of neurobiological processes, containing cerebrovascular dysfunction, impaired neural network connectivity, neuroinflammation, neurotransmitter imbalance, and altered brain metabolism [4, 7].

Sevoflurane, considered as a safe reagent in clinical use, is a common anesthetic for adults and children, and is characterized by low blood gas coefficient, quick action and quick recovery [8]. However, recent studied have shown that sevoflurane plays neurotoxic functions in the central nervous system, including cognitive dysfunction and abnormal behavior [9, 10]. A randomized clinical trial study of Mei et al. [11] showed that 23.3% patients (24/103) anesthetized with sevoflurane occurred POCD. Another study has found that compared with propofol, sevoflurane anesthesia was in connection with a higher incidence of POCD in the elderly patient population [12]. Besides, sevoflurane inhalation anesthesia could contribute to POCD by dose dependence, which may be involved in the pathway activation of inflammation and apoptosis [13]. However, the pathogenesis and molecular mechanisms of POCD induced by sevoflurane remain unclear.

Exosomes, as the carrier of bioactive molecules such as RNA, DNA, and proteins, range from 50 to 200 nm in size, and are an important tool for cell-to-cell communications [14]. A previous study indicated that the release of plasma-derived exosomal α-synuclein could induce POCD of geriatric hip fracture patients [15], which implied the importance of exosomes in delirium. MircoRNAs (miRNAs) with 17-26nt length can regulate gene expression by suppressing and degrading target gene translation [16, 17]. Compared with the non-exosome-related miRNAs, exosomes-associated miRNAs are more stable, and more resistant to RNase enzyme activity [18]. Furthermore, more and more evidence suggested that miRNAs determined in the body fluids (such as blood and urine) are crucial regulators in cell communications, as well as play essential roles in various physiological and pathological processes and homeostasis [19, 20]. Chen et al. [21] found that POCD occurred in 63 of 370 elderly patients with gastric cancer 7 days after surgery, and serum miR-210 expression was an independent risk factor of POCD. However, the roles of exosomal miRNAs in the occurrence and progression of POCD are still unknow.

Recently, it has been reported that peripheral inflammatory stimulation can lead to activation of microglia, and the aggregation and activation of microglia are found to be closely concerned with the pathogenesis of nervous system diseases, like POCD and Alzheimer’s disease [22]. Activated microglia can heighten oxidative stress and induce cell death pathway through releasing various inflammatory factors, thereby accelerating neurodegeneration (such as POCD) [23]. Additionally, human microglia HMC3 cells have been widely applied for neurodegenerative diseases research, due to the small number and the time-consuming technology of primary microglia cultures [24]. Therefore, exosomes were firstly isolated from the plasma of sevoflurane-treated POCD and non-POCD patients, and then sent for small RNA sequencing. After sequencing and analyzing, miR-548-5p was enriched in the POCD-associated plasma-derived exosomes, as well as was chosen for subsequent study. Finally, the effects and related mechanisms of POCD-derived exosomal miR-584-5p on human microglia HMC3 cells were further explored. These results will provide valuable clues for the discovery of new therapeutic targets and pathways to manage sevoflurane-induced POCD.

## RESULTS

### Clinical information

In this study, 20 patients from the patients with general anesthesia were selected for further study, and Table 1 showed the basic clinical information of the selected 20 subjects.

**Table 1:** Basic clinical information of the selected 20 patients.

**Index**	**Non-POCD (n = 10)**	**POCD (n = 10)**
Age	70.20 ± 3.49	77.30 ± 6.77
Male	N = 5	N = 5
Female	N = 5	N = 5
BMI	20.82 ± 2.38	20.22 ± 2.42
Education	8.40 ± 2.37	10.70 ± 3.27
MMSE	21.90 ± 3.60	19.60 ± 3.98
Preoperative nerve block	N = 7	N = 1

POCD, post operative cognitive dysfunction; BMI, body mass index; MMSE, mini-mental state examination.

### Identification of the isolated exosomes

**Figure 1:**
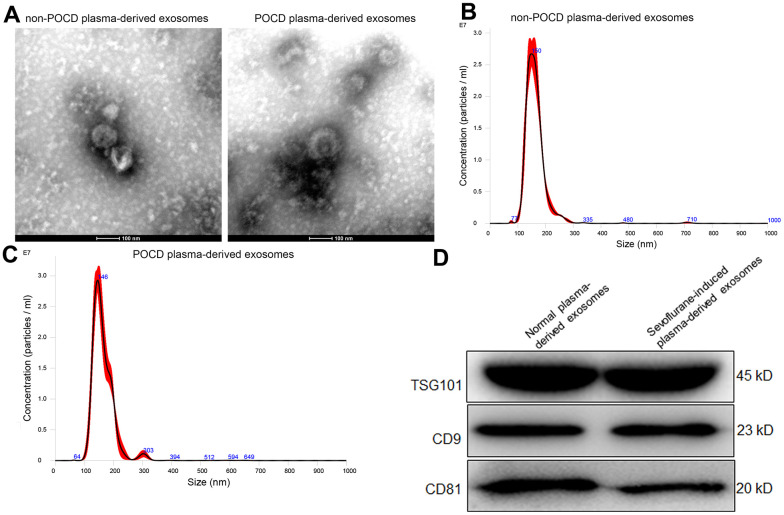
**Characterization of exosomes isolated from the plasma of sevoflurane-induced post operative cognitive dysfunction (POCD) patients or non-POCD patients.** (**A**) The morphology of exosomes was visualized using a transmission electron microscopy. A NanoSight NS300 particle size analyser was used to analyze the particle size of the exosomes isolated from the plasma of sevoflurane-induced non-POCD patients (**B**) and POCD patients (**C**). (**D**) The expression of exosomes-specific markers TSG101, CD9 and CD81 examined by western blot.

Exosomes were extracted from the plasma of POCD patients induced by sevoflurane or non-POCD patients, and then were identified by western blot, TEM, and NTA. It was found that the matters extracted from the plasma of POCD or non-POCD patients were both displayed morphology of nearly round of cup-shaped with a diameter of about 100 nm (Figure 1A). The results of NTA displayed that the main peaks of the POCD-associated exosomes and non-POCD-associated exosomes were respectively 146 nm and 150 nm (Figure 1B, 1C), which were coincided with the size distribution of exosomes described as previously [25]. Besides, CD9, TSG101, and CD81, which are the exosomes-specific markers, were all expressed in the POCD-associated exosomes and non-POCD-associated exosomes (Figure 1D). Combined with the results of western blot, TEM and NTA, we can find that the exosomes were successfully extracted from the plasma of the POCD or non-POCD patients induced by sevoflurane.

### Screen of DE-miRNAs between the sevoflurane-induced POCD-associated and non-POCD-associated exosomes and functional analyses

**Figure 2:**
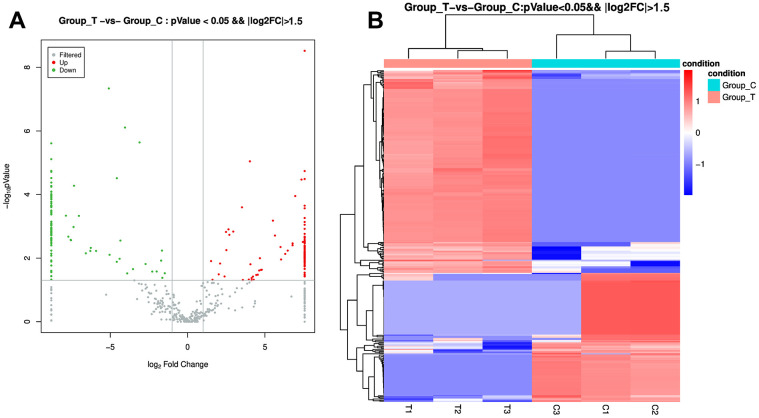
**Identification of differentially expressed microRNAs (DE-miRNAs) between the POCD-associated exosomes and non-POCD-associated exosomes.** (**A**) The volcano figure of DE-miRNAs with the criteria of |log_2_ FC| > 1.5, and P value < 0.05. (**B**) The bidirectional hierarchical cluster analysis of these DE-miRNAs.

To comprehend the underlying molecular mechanisms of sevoflurane-induced POCD progression, the exosomes from the POCD and non-POCD patients were then subjected for small RNA sequencing. Totally, 301 DE-miRNAs, including 184 up-regulated miRNAs and 117 down-regulated miRNAs, were observed between the sevoflurane-induced POCD-associated and non-POCD-associated exosomes (Figure 2A and Supplementary Table 1). Then, the 301 DE-miRNAs were bidirectional hierarchical clustered, and the results showed that these DE-miRNAs were able to well differentiate the POCD-associated exosomes (T group) and non-POCD-associated exosomes (C group, Figure 2B).

**Figure 3:**
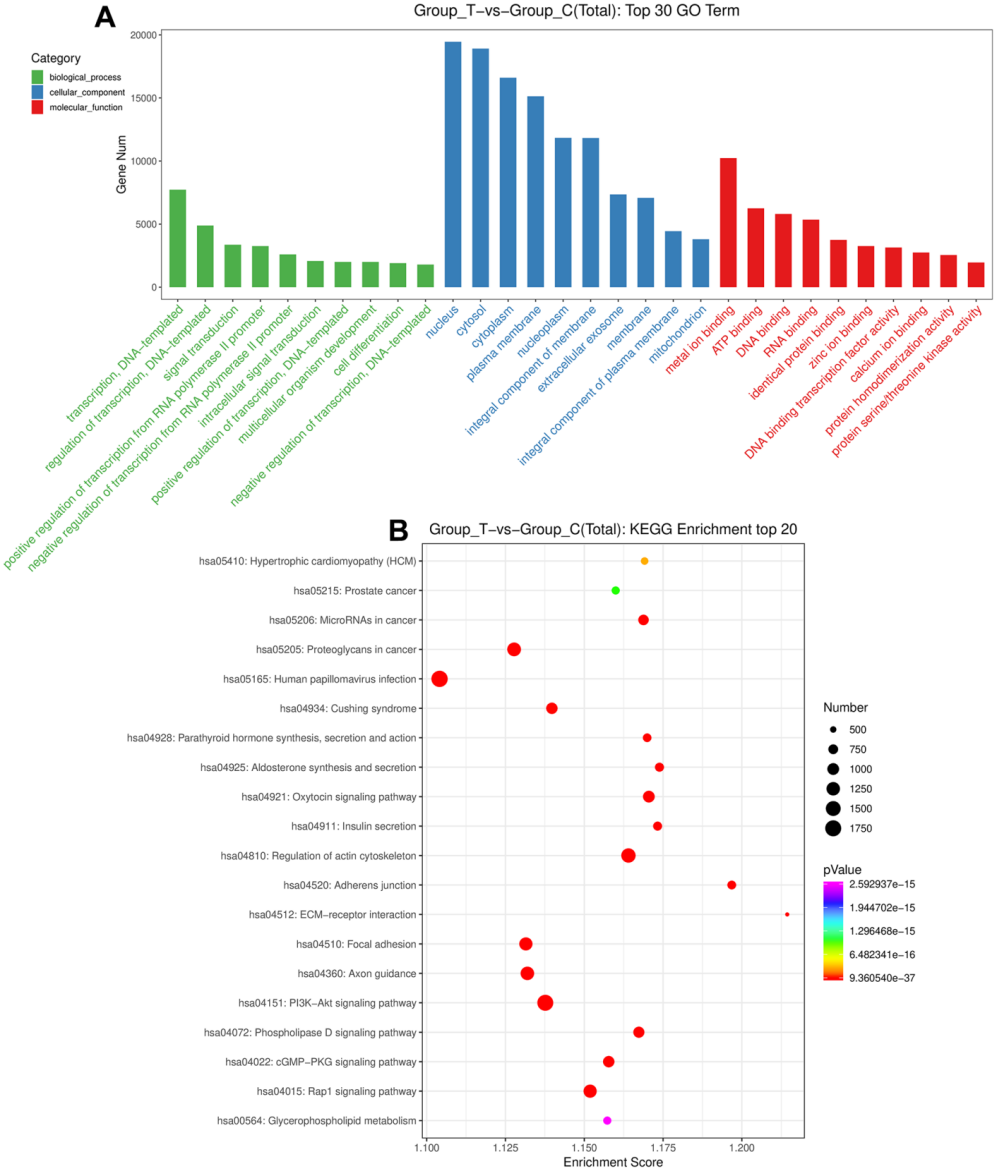
**Functional analyses of the identified DE-miRNAs.** (**A**) The top 30 Gene Ontology terms of these DE-miRNAs in biological process, cellular component and molecular function. (**B**) The top 20 Kyoto Encyclopedia of Genes and Genomes pathways enrichment of these DE-miRNAs.

After that, the DE-miRNAs were used to predict the target genes, and 84604 mRNAs were found for functional analyses. Following *P* value < 0.05, a total of 3577 Go terms, as well as 121 KEGG pathways were significantly enriched by these genes (Supplementary Tables 2, 3). It is obvious that these DE-miRNAs were significantly enriched in “multicellular organism development”, “positive regulation of transcription, DNA-templated”, and “intracellular signal transduction” of biological process GO terms; as well as in “mitochondrion”, “integral component of membrane” and “extracellular exosome” of cellular component GO terms; and in “zinc ion binding”, “protein homodimerization activity”, “ATP binding”, and “calcium ion binding” of molecular function GO terms (Figure 3A). Furthermore, these DE-miRNAs were also significantly related to “Oxytocin signaling pathway”, “ECM-receptor interaction”, “Axon guidance”, “PI3K-Akt signaling pathway”, “regulation of actin cytoskeleton”, “phospholipase D signaling pathway”, “cGMP-PKG signaling pathway”, “Glycerophospholipid metabolism”, as well as “Rap1 signaling pathway” (Figure 3B).

### Validation of sequencing data by RT-qPCR

**Figure 4:**
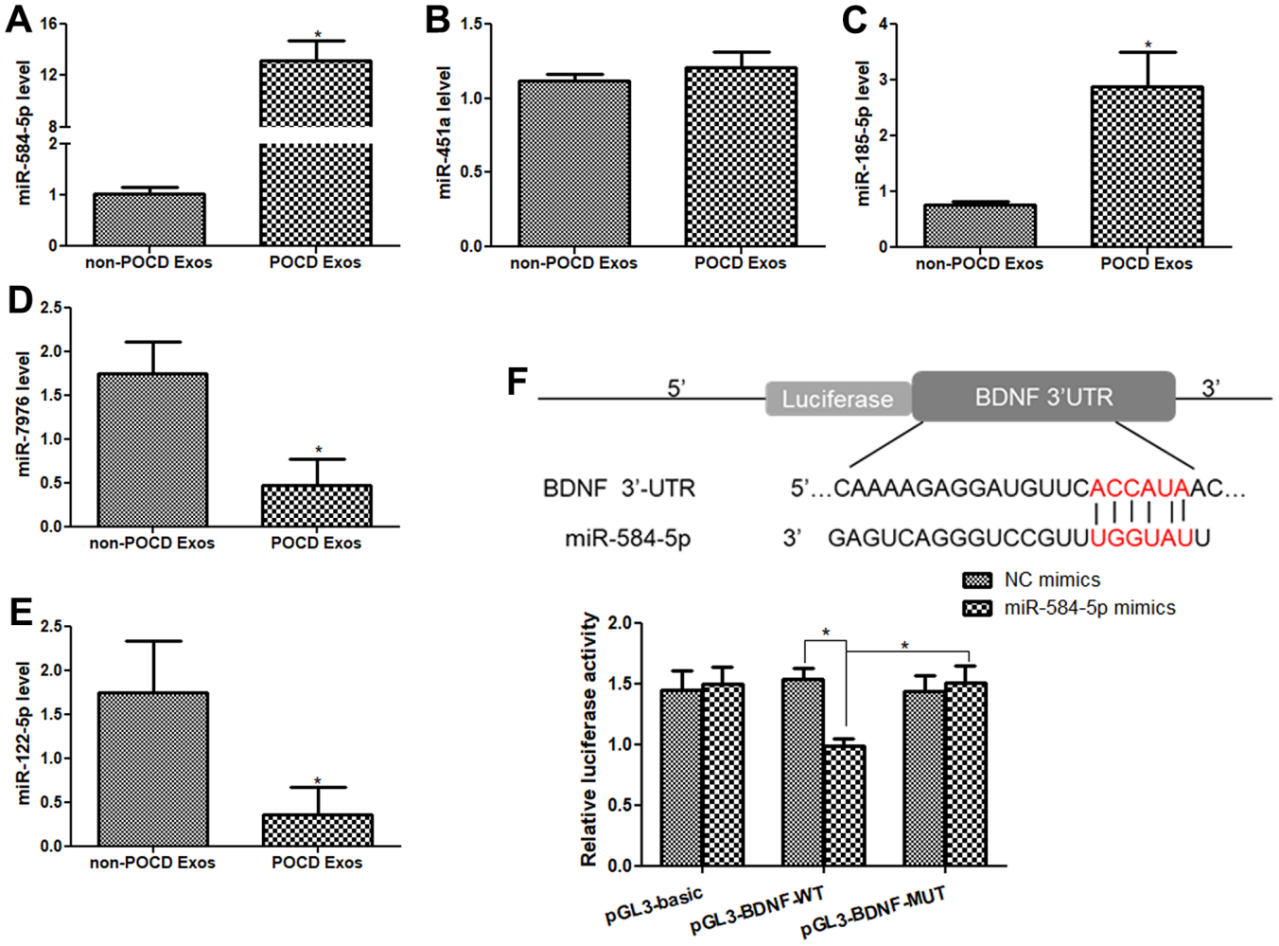
**Validation of sequencing result by real-time quantitative PCR (RT-qPCR), and *BDNF* was the target gene of miR-584-5p.** The mRNA levels of miR-584-5p (**A**), miR-451a (**B**), miR-185-5p (**C**), miR-7976 (**D**) and miR-122-5p (**E**) in the non-POCD-derived exosomes and POCD-derived exosomes. N = 10. *: *P* < 0.05, compared with the non-POCD-derived exosomes. (**F**) An online tool TargetScan Human 7.1 was applied to predict that BDNF was the target gene of miR-584-5p, and dual-luciferase reporter gene assay showed that *BDNF* was confirmed to bind with miR-584-5p. *: *P* < 0.05.

We then selected three up-regulated miRNAs (miR-451a, miR185-5p, and miR-584-5p), as well as two down-regulated miRNAs (miR-7976, and miR-122-5p) for further validation. Relative to the non-POCD plasma-derived exosomes, miR-584-5p level was evidently increased in the sevoflurane-induced POCD plasma-derived exosomes (*P* < 0.05, Figure 4A). The miR-451a level in the POCD-associated exosomes and non-POCD-associated exosomes had no significant difference (*P* > 0.05, Figure 4B). The trend of the miR-185-5p level in the different groups was similar with that of the miR-584-5p level (Figure 4C). For miR-122-5p and miR-7976, their levels were markedly decreased in the POCD-derived exosomes (*P* < 0.05) compared to the non-POCD-derived exosomes (Figure 4D, 4E). All the outcomes demonstrated that the coincidence rate between RT-qPCR results and sequencing analysis was 80% (4/5×100%), which implied that the sequencing results were relatively highly reliable. Due to the relative high level of miR-584-5p in the POCD plasma-derived exosomes, hsa-miR-584-5p was selected for following experiments.

### BDNF was the target gene of miR-584-5p

TargetScan Human 7.1 found that the 3’-UTR of *BDNF* had the binding site of miR-584-5p, so we predicted that *BDNF* may be the target of miR-584-5p (Figure 4F). Thereafter, to confirm this hypothesis, dual-luciferase reporter gene assay was performed. In the pGL3-basic plasmid, the relative luciferase activity in the NC mimic and miR-584-5p mimics displayed no significant difference(*P* > 0.05, Figure 4F). In the pGL3-BDNF-WT plasmid, relative to the NC mimics, the relative luciferase activity in the cells after transfected with miR-584-5p mimics was significantly reduced (*P* < 0.05); but the relative luciferase activity was restored to a similar of NC mimics after *BDNF* mutated (in the pGL3-BDNF-MUT plasmid, *P* > 0.05, Figure 4F). The results suggested that *BDNF* could directly bind with miR-584-5p.

### Cellular uptake of POCD-associated exosomes and cell transfection efficiency

**Figure 5:**
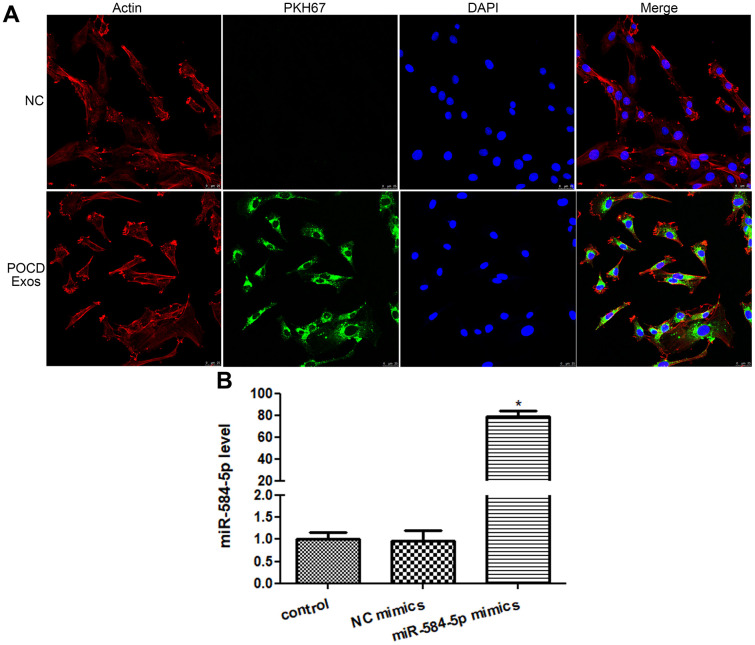
**Cellular uptake of exosomes and cell transfection efficiency.** (**A**) Sevoflurane-induced POCD-derived exosomes were labeled by PKH67 (green), and cytoskeleton of HMC3 cells was stained by Actin (red). Exosomes could be taken up by HMC3 cells after co-cultured. (**B**) The level of miR-584-5p after transfected with negative control (NC) mimics and miR-584-5p mimics. *: *P* < 0.05, compared with the control group.

The POCD-associated exosomes were labeled with PKH67 (green), as well as Actin red was stained cytoskeleton of HMC3 cells (red), and the nucleus (blue) of HMC3 cells were stained by DAPI. After 48 h of co-culture for, the intracellular green fluorescence was displayed in the HMC3 cells (Figure 5A). These manifested that the POCD-associated exosomes could be taken up by HMC3 cells.

Next, to learn the action of exosomal miR-584-5p in the growth of HMC3 cells, the HMC3 cells with miR-584-5p enrichment were constructed. There was no significant difference (*P* > 0.05) in the miR-584-5p level between the control (1.01 ± 0.16) and NC mimics (0.96 ± 0.25) groups (Figure 5B). However, after miR-584-5p mimics transfection, the miR-584-5p level reached to 79.29 ± 5.20, which was approximately 80 times that of the control group (Figure 5B). These finding revealed that the HMC3 cells with miR-584-5p enrichment were built successfully, and was able to be employed for subsequent study.

### Analyses of cell viability and apoptosis

**Figure 6:**
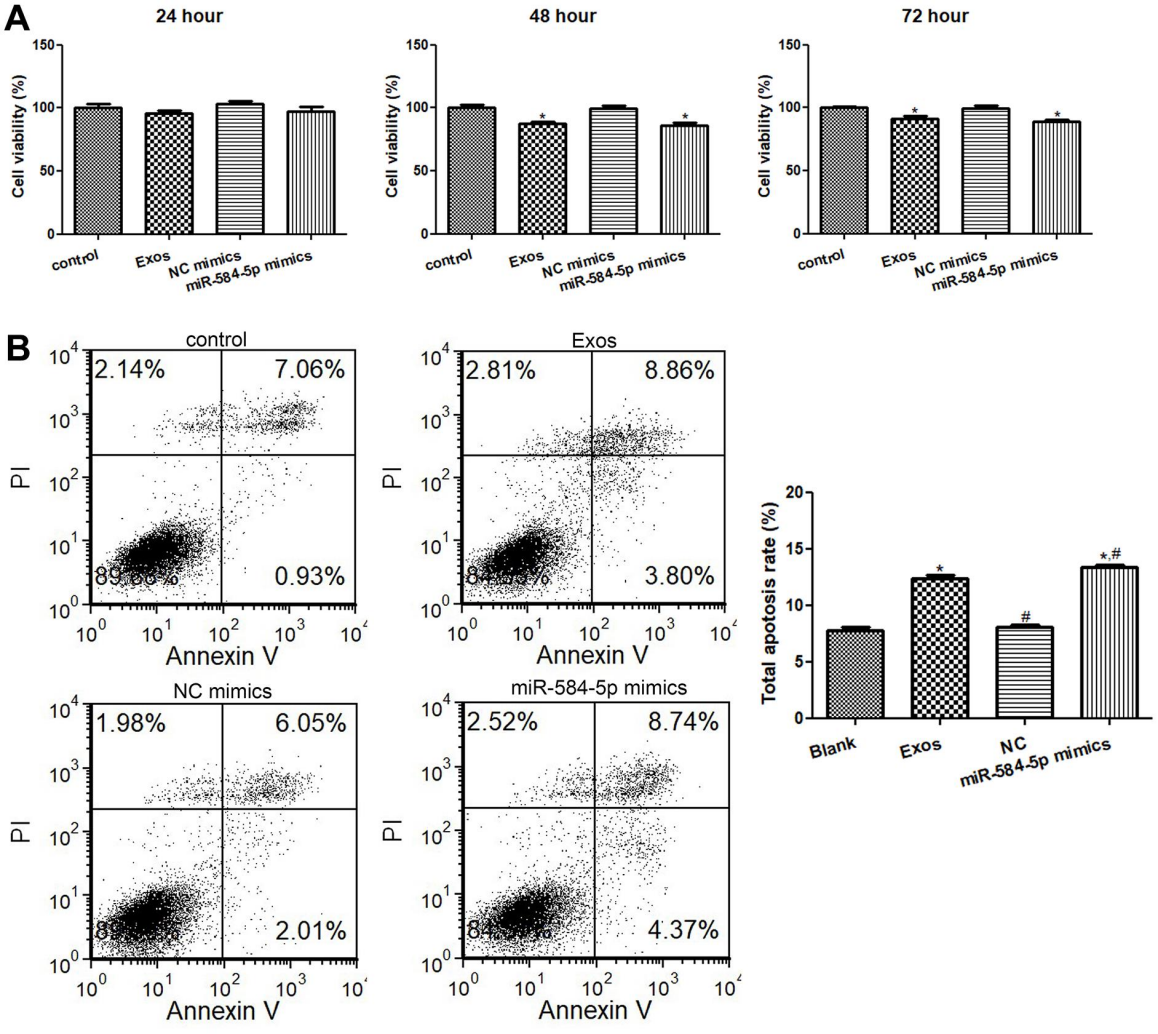
**Effects of exosomal miR-584-5p on the cell viability and apoptosis of HMC3 cells.** (**A**) The cell viability of HMC3 cells treated with POCD-derived exosomes and miR-584-5p mimics determined using Cell Counting Kit-8. (**B**) Flow cytometer was utilized to determine the cell apoptosis of HMC33 cells with different treatments. *: *P* < 0.05, compared with the control group. ^#^: *P* < 0.05, compared with the Exos group.

When the cells cultured for 24 h, 48 h and 72 h, the cell viability in the control and NC mimics groups had no obvious difference (*P* > 0.05, Figure 6A). After 24 h of culture, relative to the control group, miR-584-5p mimics transfection and exosomes treatment decreased the cell viability, but there was no significant difference (*P* > 0.05, Figure 6A). After cultured for 48 and 72 h, miR-584-5p enrichment and exosomes treatment markedly reduced the cell viability of HMC3 cells (*P* < 0.05) relative to the control cells (Figure 6A). Thus, HMC3 cells transfected/treated with miR-584-5p mimics or the POCD-derived exosomes for 48 h were selected for farther analyses.

Then, the cell apoptosis of HMC3 cells were determined. It is clear that no obvious difference in cell apoptosis was found (*P* > 0.05) between the control and NC mimics groups (Figure 6B). Relative to the control group, exosomes treatment and miR-584-5p mimics transfection both evidently elevated the total apoptosis rate (*P* < 0.05); as well as the total apoptosis rate in the cells treated with miR-584-5p enrichment was markedly higher than that in the cells with exosomes treatment (*P* < 0.05, Figure 6B). Taken together, miR-584-5p enrichment and sevoflurane-induced POCD-associated exosomes could inhibit cell viability of HMC3 cells, as well as promote their apoptosis.

### Analysis of IL-1β and TNF-α levels in the HMC3 cells

**Figure 7:**
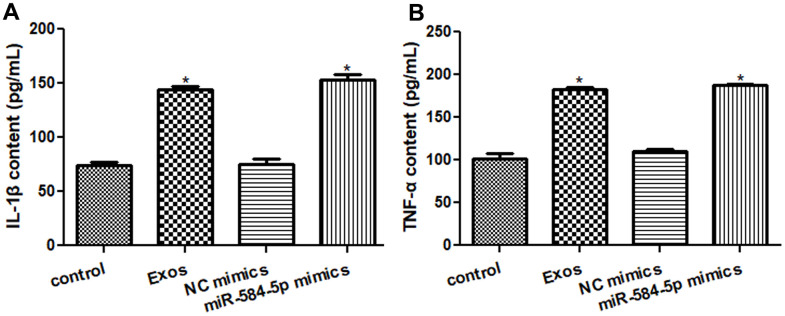
**Effects of exosomal miR-584-5p on the pro-inflammatory cytokines in HMC3 cells by enzyme-linked immunosorbent assay.** (**A**) The content of IL-1β in the HMC cells with different treatments. (**B**) The content of TNF-α in the HMC cells with different treatments. *: *P* < 0.05, compared with the control group.

After that, ELISA was applied to measure TNF-α and IL-1β contents in the HMC3 cells with different treatments. The IL-1β levels in the control as well as NC mimics groups were respectively 74.33 ± 4.72 pg/mL and 75.41 ± 8.28 pg/mL (Figure 7A); and the contents of TNF-α in the control and NC mimics were severally 101.19 ± 11.79 pg/mL and 110.08 ± 5.58 pg/mL (Figure 7B), which showed no obvious changes between the control and NC mimics. The IL-1β contents in the Exos and miR-584-5p mimics groups were 144.10 ± 4.93 pg/mL and 153.02 ± 8.43 pg/mL, respectively (Figure 7A); and the TNF-α contents in the HMC3 cells with exosomes and miR-584-5p mimics transfection were respectively 182.51 ± 4.91 pg/mL and 187.36 ± 3.12 pg/mL (Figure 7B). These showed the significant increased TNF-α and IL-1β levels in the Exos as well as miR-584-5p mimics groups than in the control cells (*P* < 0.05).

### Roles of exosomes and miR-584-5p in BDNF, TrkB, p-TrkB, Caspase 3, and IL-1β expression

**Figure 8:**
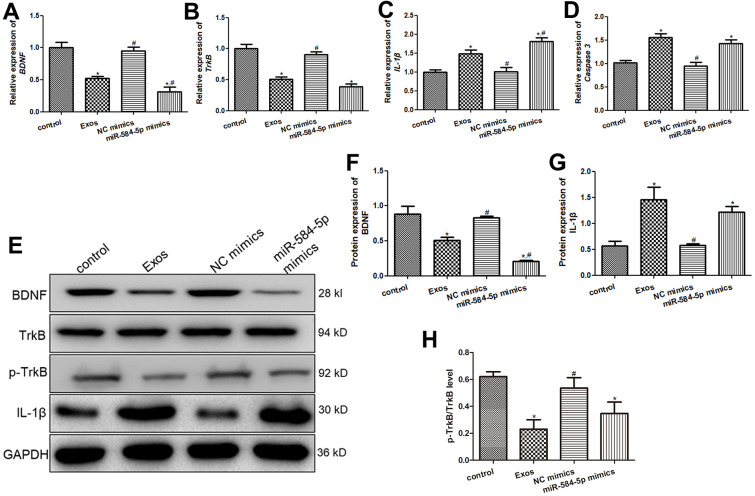
**Effects of exosomal miR-584-5p on the expression of related genes and proteins in HMC3 cells.** The mRNA expression of *BDNF* (**A**), *TrkB* (**B**), *IL-1*β (**C**) and *Caspase 3* (**D**) in the HMC3 cells after transfected with exosomes and miR-584-5p mimics using RT-qPCR. (**E**) The protein bands visualized by western blot. The protein expression levels of BDNF (**F**), IL-1β (**G**) and p-TrkB (**H**). *: *P* < 0.05, compared with the control group. ^#^: *P* < 0.05, compared with the Exos group.

Western blot and RT-qPCR were employed to detected BDNF, TrkB, p-TrkB, Caspase 3, and IL-1β expression levels in the HMC3 cells with different treatments. No significant differences in mRNA and protein expression of BDNF, TrkB or p-TrkB/TrkB, IL-1β and Caspase 3 were discovered between the NC mimics and control groups (Figure 8). Relative to the control cells, *BDNF* and *TrkB* mRNA expression were both remarkedly down-regulated in the miR-584-5p mimics and Exos groups (*P* < 0.05), and the mRNA level of *BDNF* in the cells with miR-584-5p enrichment was evidently lower than tin the cells treated with exosomes (*P* < 0.05, Figure 8A, 8B). For *Caspase 3* and *IL-1β*, their expressions were signally up-regulated after POCD-derived exosomes treatment and miR-584-5p enrichment (*P* < 0.05), relative to the control cells (Figure 8C, 8D). Then, western blot was also applied to determine protein expressions of BDNF, TrkB, p-TrkB, and IL-1β in the cells with different treatments. It is obvious that the trend of BDNF and IL-1β protein expressions in the different groups examined by western blot was similar with that of their mRNA expression measured by RT-qPCR (Figure 8E–8G). For p-TrkB/TrkB, its level was significantly reduced after treatments of POCD-derived exosomes and miR-584-5p mimics (*P* < 0.05), compared to the control cell (Figure 8E, 8H).

## DISCUSSION

POCD is defined as an acute impairment of attention and cognition, with significant associated morbidity and mortality of the elderly [26]. Inhalation of sevoflurane can cause neuronal apoptosis, cognitive disorders, and abnormal behavior, inducing to the occurrence and progression of POCD [27]. Exosomes can deliver miRNAs, lncRNAs, mRNAs, proteins, and other bioactive substances to neighboring cells or even distant cells, thus playing essential roles in the occurrence and development of diseases [14]. In the present study, we isolated the exosomes from the plasma of sevoflurane-induced POCD or non-POCD patients, and small RNA sequencing was performed. After sequencing analysis, 184 up-regulated and 117 down-regulated miRNAs were identified, and were significantly enriched in 3577 GO terms and 121 KEGG pathways (*P* < 0.05), containing”Rap1 signaling pathway”, “ECM-receptor interaction”, “phospholipase D signaling pathway”, “PI3K-Akt signaling pathway”, as well as “cGMP-PKG signaling pathway”.

Changes in hippocampal ECM (extracellular matrix) could result in the cognitive impairment related to chronic depression-like states in rats [28], and ECM-receptor interaction has been reported to participate in processes of tumor abscission, adhesion, degradation, motility and proliferation [29]. PI3K-Akt signaling pathway has been reported in the many cancers, and a study of Qiu et al. [30] showed that 5-HT(1A) receptor antagonist could improve the symptoms of delirium rats at some extent via inhibiting NLRP3 activity induced by PI3K/Akt/mTOR activation. Phospholipase D, a ubiquitous enzyme, is a conditioning agent of metabolic pathways as well as intercellular signaling [31], and phospholipase D signaling pathway is found to be closely connected with Alzheimer’s disease [32]. A previous study demonstrated that gastrodin could promote hippocampal nerve regeneration after cerebral ischemia and improve the cognitive function of mice via activating NO-cGMP-PKG signaling pathway [33]. Besides, Rap1 is essential for cell adhesion and integrin function in a variety of cell types, and Rap1 signaling pathway is reported to participate in Alzheimer’s disease [34]. Taken together, we speculate that Rap1 signaling pathway, ECM-receptor interaction, phospholipase D signaling pathway, PI3K-Akt signaling pathway, as well as cGMP-PKG signaling pathway may take part in the occurrence and development of sevoflurane-induced POCD. Nevertheless, the specific effects of these pathways on sevoflurane-induced POCD need to be further investigated.

After that, five DE-miRNAs were selected for further verification, and miR-584-5p was significantly enriched in the sevoflurane-treated POCD-associated exosomes. Further to explore the roles and potential mechanisms of exosomal miR-584-5p in POCD progression, HMC3 cells were respectively transfected or treated with miR-584-5p mimics, or sevoflurane-induced POCD-associated exosomes. In this study, we found that miR-548-5p enrichment and sevoflurane-induced POCD-associated exosomes could suppress the viability, as well as enhance apoptosis of HMC3 cells. A previous study reported that miR-124-3p in the microglial-derived exosomes could be transferred to hippocampal neurons, and improve the cognitive consequences after repetitive mild traumatic brain injury [35]. These indicated the importance of exosomal miRNAs in cognitive disorders. Additionally, many researches have reported the roles of miR-584-5p in cancers. Wei et al. [36] elaborated depletion of miR-584-5p could reduce the growth of hepatocellular carcinoma (HCC) cells via targeting KCNE2 expression, thus mediating the progression of HCC. Another study illustrated that cicPITX1/miR-584-5p/KPNB1 axis could regulate glioblastoma progression through mediating the proliferation, migration, invasion, angiogenesis, and cell cycle [37]. These reports, together with our results, we can infer that sevoflurane-induced POCD-associated exosomes delivered miR-584-5p may affect the development of POCD through inhibiting proliferation and promoting apoptosis of HMC3 cells.

Recently, cytokines dysregulation is thought to be a pivotal contributor to neurodegeneration, and subsequent cognitive disorder in delirium, which is as a consequence of the activation of a systemic inflammatory cascade [38]. A systematic review of animal experiments indicated that activation of microglial was concerned with the elevated levels of IL-1β, TNF-α, and Toll-like receptors, and could contribute to delirium [22]. TNF-α and IL-1β, pro-inflammatory cytokines, are reported to be increased in the hip fracture elderly patients with POCD [39]. Our results found that sevoflurane-caused POCD-associated exosomes and miR-584-5p enrichment significantly up-regulated TNF-α as well as IL-1β expressions, and augmented their levels in the HMC3 cells, which implied that sevoflurane-induced POCD-associated exosomes delivered miR-584-5p may promote POCD progression through up-regulating pro-inflammatory cytokines (TNF-α and IL-1β) in the HMC3 cells.

In addition, the current research showed that POCD-associated exosomes and miR-584-5p mimics down-regulated BDNF expression, and p-TrkB/TrkB level, and up-regulated *Caspase 3*. Caspase 3 is the confluence of endogenous and exogenous apoptosis pathways, and is the main executor of apoptosis [40]. Badshah et al. [41] showed that Osmotin could prevent lipopolysaccharide-induced apoptotic neurodegeneration by down-regulating PARP-1 and Caspase 3, thus improving neuroinflammation-associated memory impairment. Deregulation of BDNF/TrkB signaling pathway contributes to the pathological process of many diseases, including neurodegenerative diseases, traumatic brain injury, and cerebral ischemia, as well as down-regulation of BDNF was found to be associated with the pathogenesis of postoperative cognitive decline [42]. Another study has shown that harmine administration rose the levels of BDNF, as well as p-TrkB in both the high glucose-treated cells and the diabetic rats, and ameliorated cognitive dysfunction induced by diabetes [43]. Our study found that sevoflurane-caused POCD-associated exosomes and miR-584-5p enrichment down-regulated BDNF, and p-TrkB/TrkB, as well as dual-luciferase reporter gene assay showed that BDNF was the target gene of miR-584-5p. Therefore, it can be speculated that sevoflurane-induced POCD-associated exosomes delivered miR-584-5p may facilitate the occurrence and progression of delirium via targeting BDNF, and regulating Caspase 3 and BDNF/TrkB signaling.

However, there are some limitations in this study. First, our conclusions need to be verified in primary microglia cells and other human microglia cell lines. Second, the expanded samples are required to validate the expression of DE-miRNAs. Third, our study is preliminary, and further study should be performed to prove that whether miR-584-5p is specifically induced in sevoflurane-induced POCD via small RNA sequencing of plasma from patients treated with other anesthetic drugs. Additionally, knockdown or overexpression of BDNF or TrkB is also required to verify the growth-inhibitory roles of the miR-584-5p-BDNF/TrkB axis in POCD.

In conclusion, 301 DE-miRNAs were identified in the sevoflurane-induced POCD-associated exosomes, as well as functional analyses showed that PI3K-Akt signaling pathway, ECM-receptor interaction, phospholipase D signaling pathway, Rap1 signaling pathway, and cGMP-PKG signaling pathway may participate in sevoflurane-induced POCD progression. Additionally, POCD-associated exosomes delivered miR-584-5p could inhibit viability and promote apoptosis of HMC3 cells via targeting BDNF, as well as regulating inflammatory response and BDNF/TrkB signaling, thus accelerating the occurrence and development of sevoflurane-induced POCD. Our work helps us to improve our understanding of the pathogenesis of POCD promoted by sevoflurane, and provides foundation for miR-584-5p as well as BDNF as novel potential therapeutic targets to manage anesthetics-induced POCD.

## MATERIALS AND METHODS

### Patients and plasma collection

Totally, 275 patients aged 60-80 years old with hip replacement (ASA grade: I - III) were recruited from China-Japan Friendship Hospital, Jilin University, and 92 patients were signed the informed consent forms. However, 11 patients did not complete the test (n = 2) or dropped out of the study (n = 9). Therefore, only 81 patients were used for further study. The inclusion criteria were the patients aged 60-80 years old, the ASA grade of I-III, no history of mental illness, no history of addiction to psychotropic drugs, and no serious basic diseases. The exclusion criteria for selected patients included the patients who refused to participate and did not cooperate with the preoperative test; the patients with severe systemic diseases or ASA grade over III; the patients with long-term use of antipsychotic medications; the patients with cerebrovascular history or major neurological dysfunction; the patients diagnosed with dementia and/or severe cognitive impairment; and the patients with the scores less than the basic line based on mini-mental state examination (MMSE), as well as Montreal cognitive assessment (MoCA).

We selected ten POCD patients (T group) and ten non-POCD patients (C group) from the patients with general anesthesia, and 10 mL blood were taken from each patient. The protocols of general anesthesia were shown as follow: 4 μg/kg fentanyl, 0.2 mg/kg atracurium, and 1.2 mg/kg propofol, as well as sevoflurane (1.5%-3%) was used to keep the patients to maintain anesthesia during the surgery of hip replacement. The obtained blood was centrifuged at 4° C at 1900 g for 10 min, followed by at 3000 g for 15 min. The supernatant was collected, which was plasma.

### Isolation and identification of plasma-derived exosomes

As described as previously, exosomes were isolated from the plasma of different groups (C and T groups) under the condition of 4° C [44]. Briefly, the obtained plasma was centrifuged at 12000 g for 30 min to wipe off the cell fragments. The supernatant was filtered with a 0.22μm sterile filter to eliminate large particle vesicles above 220 nm. After centrifuged at 120000 g for 60 min, followed by 120000 g for 60 min. The sediments were resuspended with 200 μL pre-cooled PBS, which was exosomes. Following, a transmission electron microscope ((TEM, JEOL LTD, USA) [45], a NanoSight NS300 particle size analyser (NTA, Malvern Panalytical, UK) [46], and western blot (CD9, CD81 and TSG101, 1:500) [47] were used to characterize the isolated exosomes.

### Small RNA sequencing

The isolated exosomes from the plasma of T and C groups were submitted to Yanzai Biotechnology (Shanghai) Co. Ltd (Shanghai, China) for small RNA sequencing. A mirVANA miRNA Isolation kit (Takara, Beijing, China) was used to extracted total RNA from the exosomes. After determining the concentration and quality, the total RNA was sequenced using Illumina Miseq platform. After data pre-processing, clean data were aligned to the Rfam database, Repbase database and miRbase database to annotate miRNAs using bowtie software. After that, differential expressed miRNAs (DE-miRNAs) were identified with the thresholds of |log_2_Fold change (FC)| > 1.5, as well as *P* value < 0.05. Then, the targets of the identified DE-miRNAs were forecasted by miranda, as well as were subjected for functional analyses based on the Gene Ontology (GO) and Kyoto Encyclopedia of Genes and Genomes (KEGG) pathway databases. with *P* value < 0.05 was set as the standard of the significant enrichment.

### Co-culture of exosomes and HMC3 cells

The isolated exosomes were labeled with PKH67 using a PKH67 staining kit (green fluorescent, PKH67GL-1KT, USA). Briefly, 700 μL exosomes added to 1300 μL Diluent C were mixed with 16 μL PKH67 added to 2 mL Diluent C, and incubated for 5 min. After terminating the dyeing with 4 mL 1% BSA, the mixture was centrifuged at 4° C at 120000 g for 90 min, and the sediments were resuspended with 300 μL PBS, that is the PKH67-labeled exosomes (green).

Human microglia HMC3 cells were obtained from Procell Life Science and Technology Co. Ltd. (Wuhan, China), and were maintained in Dulbecco’s modification of Eagle’s medium (DEME, Gibco) with 10% fetal calf serum (FBS, Gibco) and 1% penicillin/streptomycin (Gibco). The HMC3 cells were inoculated into a 24-well plate, and cultured overnight. Then, 10 μL PKH67-labeled exosomes were added to the cells, and after 48 h of co-cultured, the cells were fixed with 4% paraformaldehyde for 20 min. After washing, 0.1% Triton X-100 was added for 20 min, as well as then 3% BSA was added to block for 1 h. After washing, Actin red staining solution was added to the cell in the dark for 20 min. After washing twice, mounting medium with DAPI was added to seal the slides, and a laser scanning confocal microscope (Leica Microsystems, Inc., USA) was used to photograph the cells.

### Cell transfection

Yanzai Biotechnology (Shanghai) Co. Ltd prepared and provided the negative control (NC) mimics and miR-584-5p mimics. The methods of cell transfection were described as previously [48]. Briefly, the HMC3 cells (5 × 10^4^ cells/well) were inoculated into a 24-well plate. After the HMC3 cells grew to a 70%confluence, the HMC3 cells were transfected with NC mimics and miR-584-5p mimics with Lipofectamine 2000 (Thermo Fisher Scientific) according to the protocols of manufacturer. After transfected for 6 h, the medium was changed, and cultured for another 48 h. The cells with different treatments were harvested to separate the total RNA, as well as the miR-584-5p level was measured to assess the cell transfection efficiency.

### Cell viability and apoptosis assays

A Cell Counting Kit-8 (CCK-8, Becton, Dickinson and Company, USA) was employed to determine the cell viability. The HMC3 cells were seeded into a 96-well plate, and then were divided into four groups: control group, Exos group, NC mimics group and miR-584-5p mimics group. The cells in the Exos group, NC mimics group and miR-584-5p mimics group were transfected with 10 μg plasma-derived exosomes from sevoflurane-induced POCD patients, NC mimics, as well as miR-584-5p mimics, respectively. Additionally, the HMC3 cells in the control group were without any treatment. After cultured for 24 h, 48 h, and 72 h, 10 μL CCK-8 regent was added to each well, and hatched for 2 h. Finally, a microplate reader (Thermo Fisher Scientific) was used to determine the value of OD_450 nm_.

After that, the cell apoptosis of HMC3 cells were examined using an Annexin V-FITC/PI apoptosis assay kit (Becton, Dickinson and Company). Briefly, the different cells were harvested, and resuspended in 100 μL 1 × binding buffer. Then, 5 μL Annexin V-FITC and 5 μL PI were added to stain the cells. After incubated in the dark for 15 min at room temperature, 400 μL 1× binding buffer was added, and the cell images were acquired by flow cytometer.

### Enzyme-linked immunosorbent assay (ELISA)

The cell suspension with different treatments was centrifuged at 1000 g for 10 min, followed by at 3000 g for 20 min. After that, the supernatant was collected for ELISA of IL-1β and TNF-α using their corresponding ELISA assay kit (Nanjing Jiancheng Bioengineering Institute, Nanjing, China) based on the manufacturer’s protocols.

### Dual-luciferase reporter gene assay

The target gene of miR-584-5p was forecasted by an online tool TargetScan Human 7.1 (http://www.targetscan.org/vert_71/). The dual-luciferase reporter gene assay was conducted as previous description [49]. The 3′-untranslated region (3′-UTR) sequences of brain derived neurotrophic factor (*BDNF*) were synthesized and provided by Yanzai Biotechnology (Shanghai) Co. Ltd. Besides, the pGL3-basic vector was used to construct the 3’-UTR WT/MUT *BDNF* reporter plasmid (pGL3-BDNF-WT/MUT), and 293T cells were used for cell transfection using Lipofectamine 2000. The relative light unit was determined using a dual-luciferase reporter gene assay kit (Promega, USA) based on the methods of manufacturer.

### Real-time quantitative PCR (RT-qPCR)

The total RNA was reverse transcribed into cDNA using PrimeScript™ II 1st Strand cDNA synthesis Kit (Takara). For the levels of miRNAs, stem ring method was used, and U6 was used as the housekeeping gene [50]. For mRNA expression, glyceraldehyde-3-phosphate dehydrogenase (*GAPDH*) was used as the reference gene. The sequences of all primers were shown in Table 2. The 2^−ΔΔCt^ method was employed to calculate miRNAs level and mRNA expression of *BDNF*, tropomycin receptor kinase B (*TrkB*), *Caspase3* and *IL-1β* [51].

**Table 2:** The sequences of all primers.

**Primer**	**Sequence (5’-3’)**
has-miR-584-5p	JH: GTCGTATCCAGTGCAGGGTCCGAGGTATTCGCACTGGATACGACC TCAGT
	F: TTATGGTTTGCCTGGG
hsa-miR-451a	JH: GTCGTATCCAGTGCAGGGTCCGAGGTATTCGCACTGGATACGACA ACTCA
	F: GCGAAACCGTTACCATTAC
hsa-miR-122-5p	JH: GTCGTATCCAGTGCAGGGTCCGAGGTATTCGCACTGGATACG ACCAAACA
	F: GCCGTGGAGTGTGACAATGG
hsa-miR-185-5p	JH: GTCGTATCCAGTGCAGGGTCCGAGGTATTCGCACTGGATACG ACTCAGGA
	F: GCTGGAGAGAAAGGCAGT
hsa-miR-7976	JH: GTCGTATCCAGTGCAGGGTCCGAGGTATTCGCACTGGATACG ACGAGCAA
	F: GCCGTGCCCTGAGACTT
downstream universal primer	R: GTGCAGGGTCCGAGGT
U6	RT: GTCGTATCCAGTGCAGGGTCCGAGGTATTCGCACTGGATACG ACAAAATATG
	F: CTCGCTTCGGCAGCACA
	R: AACGCTTCACGAATTTGCGT
BDNF	F: GGCTTGACATCATTGGCTGAC
	R: CATTGGGCCGAACTTTCTGGT
TrkB	F: TCGTGGCATTTCCGAGATTGG
	R: TCGTCAGTTTGTTTCGGGTAAA
Caspase 3	F: CATGGAAGCGAATCAATGGACT
	R: CTGTACCAGACCGAGATGTCA
Il-1β	F: ATGATGGCTTATTACAGTGGCAA
	R: GTCGGAGATTCGTAGCTGGA
GAPDH	F: TGACAACTTTGGTATCGTGGAAGG
	R: AGGCAGGGATGATGTTCTGGAGAG

### Western blot

The total protein was extracted from the cells with different treatments using RIPA lysis buffer (Beyotime Biotechnology), as well as were quantified using a BCA assay kit. Thereafter, 20 μg isolated proteins were separated by 10% SDS-PAGE, and transferred to PVEDF membranes. After blocked by 5% skim milk at 37° C for 1 h, the membranes were incubated with the primary antibodies against BDNF (1:1000, Affinity), GAPDH (1:1000, Proteintech Group, Inc.), p-TrkB (1:1000, Affinity), IL-1β(1:1000, Affinity), and TrkB (1:1000, Affinity), at 4° C overnight, respectively. Then, the membranes were incubated with the secondary antibody (1:1000, Jackson ImmunoResearch) at 37° C for 2 h. Finally, the protein bands were visualized using a Millipore ECL assay kit (Beyotime Biotechnology).

### Statistical analysis

Data were presented as mean ± standard deviation (SD). To compare the different between two groups, Student’s T test was used. For the comparison among more than two groups, one-way analysis of variance (ANOVA) followed by Tukey method were applied. Graphpad prism 5 (San Diego, CA) was employed for statistical analyses. *P* value < 0.05 was considered as statistical significance.

### Data availability statement

In this study, the sequencing data are available on the NCBI SRA database (Bioproject: PRJNA825011, https://submit.ncbi.nlm.nih.gov/about/sra/).

### Clinical trial registration

The trail was registered at Chinese Clinical Trail Registry (No. ChiCTR2200055538, http://www.chictr.org.cn/showproj.aspx?proj=148326).

## Supplementary Material

Supplementary Table 1

Supplementary Table 2

Supplementary Table 3
